# Autosomal dominant polycystic kidney disease in a family with mosaicism and hypomorphic allele

**DOI:** 10.1186/1471-2369-14-59

**Published:** 2013-03-15

**Authors:** Jana Reiterová, Jitka Štekrová, Miroslav Merta, Jaroslav Kotlas, Veronika Elišáková, Petr Lněnička, Marie Korabečná, Milada Kohoutová, Vladimír Tesař

**Affiliations:** 1 Institute of Biology and Medical Genetics of the 1st Faculty of Medicine and General Teaching Hospital, Charles University, Albertov 4, Prague 2, 128 00, Czech Republic; 2 Department of Nephrology of the 1st Faculty of Medicine and General Teaching Hospital, Charles University, U Nemocnice 2, Prague 2, 128 00, Czech Republic

**Keywords:** Autosomal dominant polycystic kidney disease, *PKD1* gene, *PKD2* gene, Hypomorphic allele, Mosaicism, Kidney transplantation

## Abstract

**Background:**

Autosomal dominant polycystic kidney disease (ADPKD) is the most common form of inherited kidney disease that results in renal failure. ADPKD is a systemic disorder with cysts and connective tissue abnormalities involving many organs. ADPKD caused by mutations in *PKD1* gene is significantly more severe than the cases caused by *PKD2* gene mutations. The large intra-familial variability of ADPKD highlights a role for genetic background.

**Case presentation:**

Here we report a case of ADPKD family initially appearing unlinked to the *PKD1* or *PKD2* loci and the influence of mosaicism and hypomorphic allele on the variability of the clinical course of the disease. A grandmother with the *PKD1* gene mutation in mosaicism (p.Val1105ArgfsX4) and with mild clinical course of ADPKD (end stage renal failure at the age of 77) seemed to have ADPKD because of *PKD2* gene mutation. On the other hand, her grandson had a severe clinical course (end stage renal disease at the age of 45) in spite of the early treatment of mild hypertension. There was found by mutational analysis of PKD genes that the severe clinical course was caused by *PKD1* gene frameshifting mutation inherited from his father and mildly affected grandmother in combination with inherited hypomorphic *PKD1* allele with described missense mutation (p.Thr2250Met) from his clinically healthy mother. The sister with two cysts and with *PKD1* hypomorphic allele became the kidney donor to her severely affected brother.

**Conclusion:**

We present the first case of ADPKD with the influence of mosaicism and hypomorphic allele of the *PKD1* gene on clinical course of ADPKD in one family. Moreover, this report illustrates the role of molecular genetic testing in assessing young related kidney donors for patients with ADPKD.

## Background

ADPKD is the most frequently inherited renal cystic disorder with an incidence between 1 in 400 and 1 in 1000. ADPKD is a systemic disorder with cysts and connective tissue abnormalities involving many organs. The progressive formation and enlargement of renal cysts causes the decline in renal function. The disease is genetically heterogeneous. Mutation either in the *PKD1* (approximately 85% of patients) or *PKD2* gene (approximately 15%) cause ADPKD, with an average age of 54.3 and 74 years, respectively, at the onset of ESRD (end stage renal disease) [1]. The greater severity of *PKD1* mutations is due to the development of more cysts at an early age, not to faster cyst growth [2]. So far, 869 different sequence variants have been reported in Polycystic Kidney Disease Mutation Database (PKDB) in the *PKD1* gene and 128 different sequence variants in the *PKD2* gene. Patients with mutations in the 5^′^ region of *PKD1* gene (until nucleotide 7812) manifest more severe disease (only 18.9% still have with adequate renal function at the age of 60 and are more likely to have intracranial aneurysms than patients with 3^′^ mutations (39.7% of whom still have adequate renal function at 60 years of age) [3]. No clear correlations were found with mutation type in both *PKD* genes or with mutation position in *PKD2* gene. The large intra-familial variability of ADPKD highlights a role for genetic background. Coinheritance of a *PKD1* hypomorphic allele in combination with an inactivating *PKD1* allele can lead to early manifestation of ADPKD [4,5]. Mosaicism can also modulate the clinical course of the disease [6,7].

Our case illustrates ADPKD initially appearing unlinked to the *PKD1* or *PKD2* loci and the influence of mosaicism and hypomorphic allele in *trans* position on the prognosis of the disease in one family. The difficulties encountered in excluding ADPKD in related potential kidney donors are also mentioned.

## Case presentation

A 45-year-old white male was examined before related preemptive renal transplantation. The patient was regularly examined by ultrasound because of positive family history of ADPKD. His 69-year-old father had ESRD at 52 years because of polycystic kidneys. Father had not well compensated hypertension many years. His grandmother with polycystic kidneys developed renal failure at 77 years. His father had one sister and one brother with normal ultrasound finding at the age of 40.

ADPKD in the patient was first diagnosed on ultrasound at the age of 20. At this age he suffered from repeated renal colic caused by urate concrements. The stones passed spontaneously after hydration. He was on antihypertensive drugs ACE inhibitor and AT1 receptor blocker because of mild hypertension since the age of 25. The blood pressure was well compensated (repeatedly below 130/80 mm Hg). There was a mild dilatation of ascending aorta and mild mitral valve insufficiency on echocardiography. The renal function started to decline at the age of 30, with ESRD reached at the age of 45. His 40-year-old sister volunteered herself as a potential kidney donor. Results of her blood group and tissue-type identified her as a suitable donor with an optimal HLA match and negative cross-match. However an ultrasound scan revealed 2 cysts in her left kidney.

The paternal grandmother developed renal failure at 77 years and then was hemodialyzed. The diagnosis of ADPKD was based on incidental ultrasound finding of renal and hepatic cysts during examination before cholecystectomy at the age of 64. Kidney size was about 16 cm in diameter, there were multiple cysts about 3 centimeters and serum creatinine was 180 μmol/l. Computed tomography or magnetic resonance were not performed. Moderate renal insufficiency was present.

## Methods and results

### Molecular analysis

**Figure 1:**
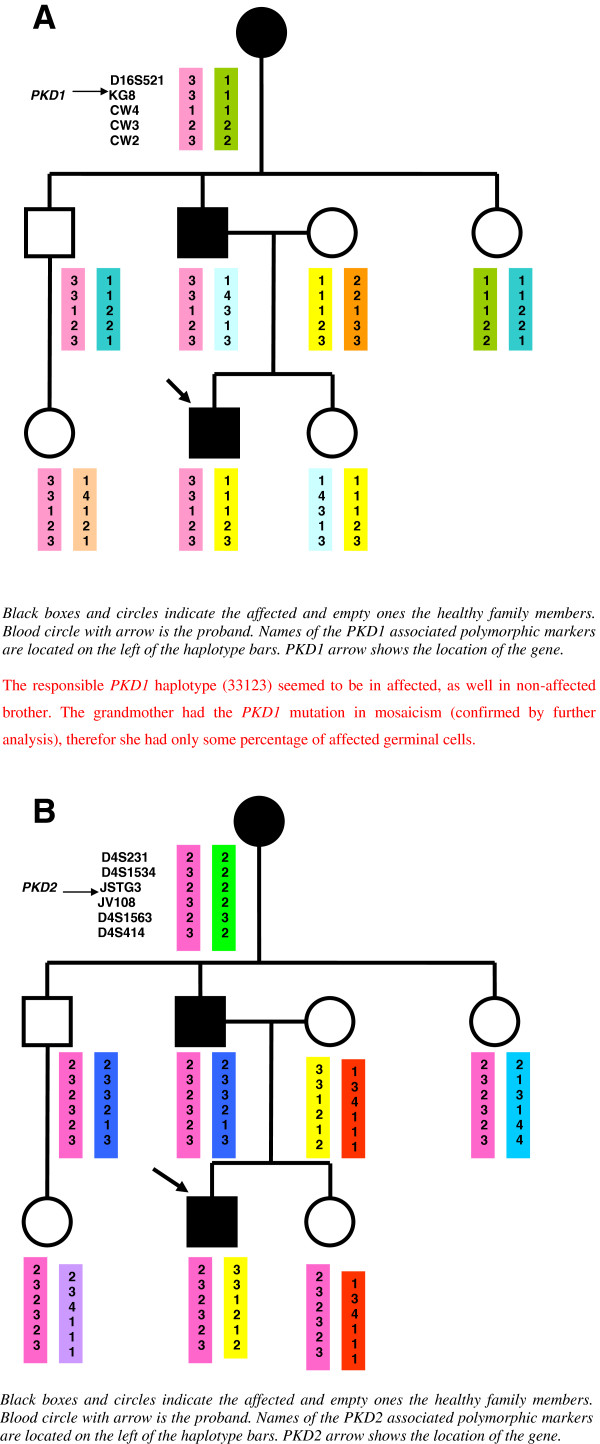
**A. Linkage analysis for the PKD1 locus. B. **Linkage analysis for the PKD2 locus.

Genomic DNA were isolated from peripheral blood lymphocytes by the salting-out procedure [8]. Linkage analysis was performed, using a panel of five and six CA-repeat flanking markers for chromosome 16 and chromosome 4, respectively, which carry the *PKD1* and *PKD2* genes. The locations of the markers relative to the *PKD1* locus are shown as follows: D16S521-*PKD1*-KG8-CW4-CW3-CW2. The locations of the markers relative to the *PKD2* locus are shown as follows:D4S231-D4S1534-JSTG3-*PKD2*-JV108-D4S1563-D4S414. Genotyping was performed by GelStar labeling of the PCR products and analyzed by PAGE (polyacrylamide gel electrophoresis). Haplotypes were constructed by hand. As markers are on both ends of the *PKD* genes, the recombination can be excluded. The haplotype analysis for the *PKD1* locus identified that the patient inherited the same haplotype as his unaffected uncle and for the *PKD2* locus the same haplotype as his unaffected uncle and aunt. The uncle and the aunt had normal finding on renal ultrasound at the age of 40 years, so the ADPKD seemed to be excluded. If we had considered only the clinical findings and linkage analysis, we would have concluded that linkage to both *PKD* genes was excluded in the family (Figure 1).

Although the haplotype with both *PKD1* and *PKD2* can be constructed to show that the affected proband can share an intact haplotype at both loci with his affected grandmother (and of course with his affected father), the inheritance of the same haplotype in the unaffected uncle (around the *PKD1* locus) or unaffected uncle and aunt (around the *PKD2* locus) could be due to non-paternity. It has been proven by means of forensic genetics that all children of the paternal grandmother are fullsiblings.

Firstly the mutational analysis of the *PKD2* gene was therefore employed in order to identify the causative mutation in the grandmother with mild clinical course. However, no mutation was found by mutational analysis of the *PKD2* gene in the grandmother who died at the age of 80.

We have used long-range PCR (LR-PCR) strategy folowed by nested PCR for mutational analysis of the *PKD1* gene [9]. Sequences of primers for LR PCR of the duplicated region of *PKD1* gene (L1 - L8; exons 1 – 34) and conditions are described in detail by Phakdeekitcharoen B et al. [10]. Sequences of primers for LR PCR of the non-duplicated region of *PKD1* gene (L9; exons 35 – 46; size 5033 bp) were: 1. L9F 5^′^ CTG TGA CCC AAG AGG CTC AAG AAA 3^′^, 2 L9R 5^′^ TGA CAC GAG ACA CAC AGT GAG ACG 3^′^. LR-PCR amplifications were performed in a total volume of 50 μl of the mixture containing 300 ng of genomic DNA, 3.75 U of Expand long template enzyme mix (Roche Applied Science).), 1× Expand long template buffer 1 with 1.75 mM MgCl_2_, 350 μM dNTP and 15 pmol of each primer. LR-PCR amplification were performed: 5 min at 98°C, followed by 35 cycles of a two-step protocol that consisted of denaturation at 95°C for 30 s, alternating with an amplicon–specific annealing and extension period for 7 min at 64°C for 70°C, followed by a final extension at 72°C for 10 min by using MyCycle PCR system (BioRad]. Using an aliquot of the LR PCR product as a template (fragments were diluted 1:200–1:500], nested PCR were performed to produce fragments which were subsequently analyzed by High Resolution Melting (HRM). Primers for nested PCR fragments were designed based on published DNA sequence (accession number L39891).

The coding region and intron-exon boundaries of the of the *PKD1* gene were screened on the whole 71 nested PCR. Sequences of primers and conditions of nested PCR are available upon request. Nested PCR were performed by using Light Cycler 480 (Roche Applied Science) in a total volume of 10 μl of the mixture containing 1 μl of diluted LR product, 1 × Light Cycle 480 High resolution melting master mix (Roche Applied Science) and 5 pmol of each primer. Nested PCR amplicons were analyzed with Light Cycle 480 Gene Scanning Software (Roche Applied Science). This software analyzes the high-resolution melting curve data to identify changes in the shape of the curve that indicate both homozygous and heterozygous allelic variants in a sample. The Gene Scanning Software generates a difference plot by subtracting the curves from a reference curve and automatically groups samples with similar melting curves [11]. All nested PCR fragments showing aberrant melting curves on HRM were sequenced in both directions using an automatic fluorescent genetic analyzer (ABI Prism™ 3130 Genetic Analyzer; PE Applied Biosystems) in accordance with the manufacturer's instructions.

**Figure 2:**
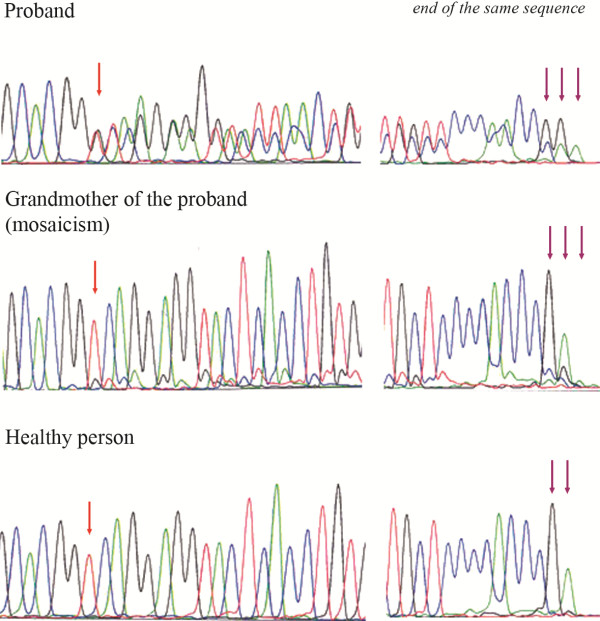
**Sequencing pattern of the part of the exon 15 (reverse strand) in proband with heterozygous mutation c.3312dupC (p.Val1105ArgfsX4) of the *PKD1 *gene, in grandmother with mosaicism and in healthy person.**

Since the linkage analysis was not informative and mutational analysis of the *PKD2* gene in the grandmother was negative, the mutational analysis of the *PKD1* gene was performed in attempt to identify the causative mutation in the patient before related kidney transplantation. The unique frameshifting mutation in the *PKD1* gene p.Val1105ArgfsX4 (c.3312dupC) (new mutation) was identified in the 45-year-old proband and in his 69-year-old father (Figure 2). The mutation p.Val1105ArgfsX4 was not present in the sister of the proband who had two cysts on a kidney on ultrasound at 40 years. Related preemptive transplantation from the sister was done.

The above described mutation was suggested to be present as a germinal and a somatic mosaic in the grandmother with mild clinical course of ADPKD. The mutation was visible in the grandmother´s DNA sample, the mutant allele was at a lower level than that found in the patient. DNA for quantitative assay of mutated allele dosage from buccal cells was not available from grandmother. Neither DNA samples nor ultrasound finding of kidneys of the parents of grandmother were available, as well. But her father died at the age of 72 because of lung cancer and her mother died at the age of 89. Finally, real time PCR using allele specific primers was performed on LightCycler 480 (Roche) to quantify the somatic mosaic in the grandmother (for mutation in the *PKD1* gene p.Val1105ArgfsX4). Using an aliquot of the LR PCR product as a template (fragments werediluted 1:200–1:500), nested PCR was performed to produce fragmentswhich were subsequently analyzed by High Resolution Melting (HRM).Forward primer for mutated allele R39M1F 5´ACAGGTGAGTACCTCCTGACCCG3´, forward primer for normal allele R39NF 5´ACAGGTGAGTACCTCCTGACCG3´ and one reverse primer R39NR 5´CCGAAGTCCCACGTGTAAAG3´. Real Time PCR was performed by Light Cycler 480 (Roche Applied Science) in a total volume of 10 μl of the mixture containing 1 μl of diluted LR product, 1 × Light Cycle 480 High resolution meltingmaster mix (Roche Applied Science) and 5 pmol of each primer. RT PCR amplifications were performed: 10 min at 95°C, followed by 45 cycles of a protocol that consisted of denaturation at 95°C for 10 s, annealing at 62°C for 15 s and extension at 72°C for 20 s, followed by a final extension at 72°C for 5 min. Real TimePCR was analyzed by relative quantification using Light Cycler 480 Software (Roche Applied Science). Such analysis showed that 10% of the chromosomes in grandmother possesses aforemetioned mutation.

Later on, during further *PKD1* mutation analysis, the described missense mutation in the *PKD1* gene p.Thr2250Met (c.6749C > T) was identified in the proband. Surprisingly this mutation was inherited from his clinically healthy 70-year-old mother (kidneys on ultrasound without cysts, normal renal function). The proband inherited the frameshift mutation p.Val1105ArgfsX4 from his affected father and the missense mutation p.Thr2250Met from his mother, so the mutations had to be in trans-position.

Because renal ultrasound on the mother was normal, a CT scan was performed, showing two small cysts. Moreover this missense mutation was identified in his sister who was a donor of the kidney. We can speculate that this missense mutation could contribute to the development of two renal cysts in the sister.

## Discussion

The family initially appearing unlinked to the *PKD1* gene and *PKD2* gene is presented. Firstly the family was considered to be probably *PKD3* family. A definitive, predictive testing for the sister of the proband before preemptive kidney transplantation was impossible from linkage analysis. The exclusion of ADPKD in any prospective kidney donor in families with ADPKD is vital because of the risk of potential living donors developing the polycystic disease themselves.

At least one family member with ESRD at or after 70 years of age is highly predictive of *PKD2* mutations [12]. Because of mild clinical course of ADPKD in grandmother (ESRD at the of 77) the mutational analysis of the *PKD2* gene was first done. No mutation was detected by heteroduplex analysis and direct sequencing.

The mutational analysis of the *PKD1* gene in the patient with advanced chronic renal insufficiency was therefore employed in attempt to identify the causative mutation. The frameshifting mutation p.Val1105ArgfsX4 in the *PKD1* gene was identified in the patient and was not present in a potential donor, in his sister. In spite of the presence of two cysts on ultrasound of the 40-year-old sister the preemptive kidney transplantation was undertaken. Renal transplantation is the optimal treatment for ADPKD and the benefits of preemptive transplantation have long been recognized.

The grandmother must have been a somatic and germinal mosaic for the frameshifting mutation p.Val1105ArgfsX4 in the *PKD1* gene and the father inherited this mutation from her and transferred the affected allele to his son. Approximately 10% of ADPKD families have a negative family history, and in several cases de novo mutations have been revealed [13]. Some of the new cases have been shown to occur as a mosaic [6,7]. As mosaicism would not normally be detected from mutation screening, a significant proportion of de novo cases in mosaic form can be missed. Later, the possible mutation p.Thr2250Met in sister was identified. The risk of cyst development in donor-sister, as well as in the graft in brother is probably a little higher. We can speculate that more cysts will develop but hopefully the renal involvement will be mild. The development of possible cysts in donor sister, as well in proband, will be regularly examined.

The gene *PKD1* is highly polymorphic with about 10 neutral variants found per patient from sequencing [14]. The significance of missense mutation p.Thr2250Met in exon 15 is not unambiguous. Missense mutation p.Thr2250Met was found in three different families in France [15]. Firstly, this change was reflected in both the Grantham matrix score and the more formal analysis as likely pathogenic mutation Group C in the ADPKD Mutation Database (http://pkdb.mayo.edu). Thereafter, this change was reclassified as likely neutral in this database. On the other hand, p.Thr2250Met mutation is described as disease causing mutation with medium risk in Human Gene Mutation Database (HGMD) Professional 2012.3. p.Thr2250Met mutation in homozygosity and in combination with *SEC63* gene mutations contributed to severe phenotype in a patient with hepatic cysts in comparison with his relatives who had p.Thr2250Met mutation in heterozygous state [16]. This last finding supports the influence of this allele on clinical course.

This allele in heterozygosity in the 40-year-old sister of the proband was associated with only occasional cyst development, the 70-year-old mother with the same change had no cysts on ultrasound. It seems that this likely pathogenic missense mutation is more likely an incompletely penetrant allele. The allele alone may result in mild cystic disease. Probably this change is only associated with a handful of renal cysts by middle age in some cases. This allele could contribute to the earlier ESRD in the proband (45 years) in comparison with his father (52 years). The difference in the ages of ESRD was only 7 years, however, father of the proband had severe hypertension that was not well compensated. Hypertension is clearly associated with more severe ADPKD irrespective of genetic background.

## Conclusions

In conclusion, we describe first an ADPKD family with variable clinical course caused by mosaicism in one affected family member and by an incompletely penetrant allele in others. The most severe clinical case in the family was caused by combination of the *PKD1* gene frameshifting mutation and hypomorphic *PKD1* allele. Moreover, this report illustrates the role of molecular genetic testing in assessing young related kidney donors for patients with ADPKD.

## Consent

The study was approved by Ethical Committee of General Teaching Hospital in Prague. Written informed consent was obtained from the patient for publication of this case report and any accompanying images. A copy of the written consent is available for review by the Editor-in-Chief of this journal.

## Abbreviations

ADPKD: Autosomal dominant polycystic kidney disease; ESRD: End stage renal disease; LR PCR: Long range polymerase chain reaction; HRM: High resolution melting; CT: Computed tomography; ACE inhibitors: Angiotensin converting enzyme; AT1 receptor blockers: Blockers for type 1 receptors of angiotension II.

## Competing interests

The authors declare that they have no competing interests.

## Authors’ contributions

JR, MM, VT participated in the conception and the design of the study, examined the patients, collected data relevant to this case report and made the clinical diagnosis. JS, VE, PN, MK designed, performed and interpreted the molecular evaluations. JK and MK consulted the patients as clinical geneticists and helped with examination of relatives. All authors read and approved the final manuscript.

## Pre-publication history

The pre-publication history for this paper can be accessed here:

http://www.biomedcentral.com/1471-2369/14/59/prepub
